# State Examination and Public Health

**Published:** 1894-05

**Authors:** 


BUFFALO MEDICAL AND SURGICAL JOURNAL

A MONTHLY REVIEW OF MEDICINE AND SURGERY.

*EDITORS:*

THOMAS LOTHROP, M. D. - - WM. WARREN POTTER, M. D.

All communications, whether of a literary or business nature, should be addressed to the managing editor: 284 Franklin Street, Buffalo, N. Y.

Vol. XXXIII. MAY, 1894. No. 10.

### STATE EXAMINATION AND PUBLIC HEALTH.

The friends of higher medical education and of state examination and license—and the same are rapidly becoming the people—have frequently been called upon to listen to the managers of our medical faculties of a too common class, justifying the graduation of the medical ignoramus upon the plea that he was a good enough doctor for the country.

Of course, they say, we don't expect him to stay in the city, in fact, we would not be proud of him as a neighbor, but he will do for the country. Surely, you do not expect a man with a trained and cultivated mind, with a full and thorough knowledge of medicine in all its branches, to bury himself in one of our frontier country villages ? And the speciousness and plausibility of this pleading prevailed mightily with the more venal and thoughtless legislator. Hence the prolonged contest that each of our commonwealths has had to get back the privilege of examining and licensing their doctors ; and so, for many years, the work of graduating the medical ignoramus for country use went on exasperatingly.

How inimical to public health and how utterly out of place is the ignorant, careless and incompetent medical man of the country we are coming to realize, thanks to the converging lights of bacteriology and sanitary science. In the thorough illumination of these specialties the harmless and obscure ways of the humble country practitioner are found to be of vital import to public health, and too frequently his oversights are fraught with enormous death-dealing potentiality.

We have only to refer to our health reports and to the consensus of intelligent medical opinion the world over, as to the communicability and the actual communication of tuberculosis, typhoid fever, scarlet fever and diphtheria by means of food supplies—water, milk and other foods of habitual and general use—in order to make clear the vital importance to public health of having only competent medical men on guard, at least, in those regions from whence comes these foods so important for the support of life and the preservation of health, so easy of pollution, and when polluted so destructive of health and life.

Standing at this point we see the wisdom and the absolute need of a state examination and license, and that for the most practical and important reason—the preservation of the public health.

No more practical questions are before our government—state or national—and none more vital and of all-embracing interest are likely to press for answer than the questions: How shall we preserve our springs and other sources of water supply from pollution? How shall we keep free from communicable disease our food-bearing animals? How shall we prevent the infection of various food products by the contagia of the zymotic diseases and thereby limit the spread of these diseases?

We would observe that no public-spirited citizen, legislator or sanitary officer will study seriously these practical questions without reaching the conviction that the fundamental requisite of the case, wanting which progress is impossible, is an efficient medical profession, and that if there be a suitable place and vocation for the medical ignoramus, the place is most emphatically not the country from whence we get our water, our milk and other foods, and his vocation is not the management of zymotic, infectious, communicable disease. Public health demands that for these localities the honor and responsibility of the practice of medicine shall be given only to those of the most approved competence, and that this competency be tested by the representatives of the state—the State Board of Medical Examiners—its object being the protection of the lives of our people, the defense of the public health.

The already large and rapidly increasing number of epidemics of typhoid fever, scarlet fever and diphtheria, directly due to polluted water and milk, demands the state examination and license of all practitioners of medicine, and indicates that in such examinations higher standards and severer tests will be the rule of the future.

